# Design of a hydroxyapatite-binding antimicrobial peptide with improved retention and antibacterial efficacy for oral pathogen control

**DOI:** 10.1038/srep38410

**Published:** 2016-12-02

**Authors:** Zhi-bin Huang, Xin Shi, Jing Mao, Shi-qiang Gong

**Affiliations:** 1 Department of Stomatology, Zhongshan Hospital, Xiamen University, Xiamen 361004, P. R. China; 2 Centre of Stomatology, Tongji Hospital, Tongji Medical College, Huazhong University of Science and Technology, Wuhan 430030, P.R. China

## Abstract

Controlling and reducing the formation of pathogenic biofilm on tooth surface is the key to the prevention and treatment of the biofilm-associated oral diseases. Antimicrobial peptides (AMPs), considered as possible future alternatives for conventional antibiotics, have been extensively studied for the control of bacterial infection. Due to the rapid dilution and degradation by human saliva, AMP preparations designed for oral use with longer retention and higher efficacy are in urgent need. To this end, a hydroxyapatite (HAp)-binding antimicrobial peptide (HBAMP), which is based on the fusion of a specific HAp-binding heptapeptide (HBP7) domain and a broad-spectrum antimicrobial peptide (KSLW) domain, has been developed in our laboratory. HBAMP was supposed to form a contact-active antibacterial interface on tooth surface to inhibit the formation of biofilms. In this study, we investigated its binding behaviour, antibacterial activity against bacteria in both planktonic and sessile states, enzymatic stability in human saliva, and cytocompatibility to human gingival fibroblasts (HGFs). Our findings suggest that HBAMP could adsorb on tooth surface to provide effective antibacterial activity with improved retention. This study provides a proof-of-concept on using conjugated molecules to promote antibacterial efficacy by synergistically actions of HBAMP free in solution and bound on tooth surface.

Many oral diseases are considered as chronic bacterial infectious diseases, such as tooth caries, periodontitis, and peri-implantitis^1^,^2^,^3^. Some microorganisms, such as *Streptococcus mutans, Lactobacillus acidophilus,* and *Actinomyces viscosus,* can colonize and form pathogenic plaque biofilms on the tooth surface, and have been proved to be major contributors to oral infectious diseases^2^,^3^,^4^. Effectively inhibiting the growth of planktonic pathogenic bacteria and the formation of biofilm on tooth surface is the basis for the prevention and treatment of these oral diseases^5^. Many antimicrobial preparations, such as conventional antibiotics, chlorhexidine (CHX), phenolic compounds and triclosan, can inhibit bacteria and biofilm effectively. However, extensive use of these antimicrobial agents can lead to some side-effects, such as tooth staining, calculus formation, drug resistance and gastrointestinal reactions^6^,^7^. In addition, they can provide only short-term antibacterial efficacy due to the dilution and degradation effect of human saliva. Therefore, searching for new antimicrobial molecules, which exhibit few or no side-effects and long-term retention in oral cavity, has been intensified in recent years^8^.

Being characterized by broad-spectrum antibiotic effects, high antibacterial efficacy at low concentration, and cytocompatibility to mammalian cells, both the natural and synthetic antimicrobial peptides (AMPs) have been considered as effective antibacterial agents with broad application perspectives^9^,^10^. Some specifically targeted antimicrobial peptides (STAMPs), were reported to selectively eliminate *S. mutans* while have no disruption on the normal flora in oral cavity^11^. However, the clinical application of these AMPs was limited, possibly due to the fact that oral cavity was continuously secreting saliva (0.04–0.39 ml/min)^12^, and thus making the actual concentration of antimicrobial peptides much lower than the effective concentration following saliva dilution and degradation. The strategy of immobilizing AMPs onto biomaterial surfaces through chemical coupling allows to develop a contact-active antimicrobial surface that kills microbes upon contact. However, the coupling procedures (eg. silanisation) are usually sophisticated and difficult to control^13^, and it is not feasible in dental practice to silanise tooth surface.

With the development of molecular biomimetic, many titanium (Ti) binding peptides^14^ were invented to create molecular bioconjugates that can interact with Ti surface while exhibits bioactivity of another tethered peptide. Conjugated molecules, consisting of antimicrobial and hexapeptidic Ti-binding peptides, have been shown to specifically adsorb to Ti surface and reduce biofilm formation^15^. Since the outer surface of tooth is enamel that mainly consists of hydroxyapatite (HAp), we designed a new molecular bioconjugate, named HAp-binding antimicrobial peptide (HBAMP), consisting of a broad-spectrum antimicrobial peptide (KSLW) and a HAp-binding heptapeptide, aiming to construct an antimicrobial coating on tooth surface to reduce biofilm formation. KSLW (KKVVFWVKFK) is a synthetic antimicrobial peptide reported by DeLuca PP *et al*.^16^. This synthetic peptide shows a broad range of antimicrobial activity against a number of oral pathogenic bacteria, including *S. mutans, S. sobrinus* and *L. acidophilus*^16^,^17^,^18^. *In vitro* study demonstrated that KSLW is cytocompatible to normal human gingival epithelial cells^19^. In addition, KSLW possesses merits in terms of stability in human saliva and safety in gastrointestinal tract, which are prerequisites for serving as a clinically used antimicrobial agent in oral cavity^16^. HAp-binding peptide (HBP7, NNHYLPR), isolated by bio-panning phage display random heptapeptide library, has exhibited specific affinity to enamel surface, making it a suitable molecule for tethering AMPs onto enamel surface^20^.

The purpose of this study was to investigate the binding behaviour of HBAMP to the HAp surface that served as tooth enamel analogue, as well as the antibacterial activity of HBAMP on planktonic bacteria and its effect on *S. mutans* biofilm. We also investigated its enzymatic stability in human saliva and cytotoxicity on human gingival fibroblasts (HGFs).

## Materials and Methods

### Peptides and hydroxyapatite disks

All peptides used in this study were purchased from the Sangon Biotech Co., Ltd. (Shanghai, China). Briefly, the peptides were synthesized using standard 9-fluorenylmethoxycarbonyl (Fmoc) solid-phase method^21^. Synthesized peptides were cleaved from the resin using 95% trifluoroacetic acid (TFA) with appropriate scavengers. The purity and quality were confirmed by reverse-phase high-performance liquid chromatography (HPLC)^22^. Peptide molecular mass was determined by matrix-assisted laser desorption ionization mass spectrometry. All peptides were purified to 95% and lyophilized for further use.

Dense circular HAp disks (8 mm diameter × 2 mm thick) were purchased from National Engineering Research Centre for Biomaterials (Sichuan, China). The HAp disks were polished with sandpapers (400, 800, and 1200 mesh) and washed in ethanol solution in an ultrasonic bath and sterilized by γ-irradiation prior to use.

### Bacterial strains and growth media

*Streptococcus sanguinis* (ATCC 10556), *Actinomyces viscosus* (ATCC 19246), *Streptococcus mutans* (ATCC 25175), were grown in Brain Heart Infusion (BHI; Difco, Detroit, MI, USA) culture medium. *Lactobacillus acidophilus* (ATCC 4356) was grown in de Man, Rogosa and Sharpe (MRS; Difco, Detroit, MI, USA) culture medium. BHIS (BHI with 2% sucrose) was used for growing *S. mutans* biofilm. All cultures were grown anaerobically (80% N_2_, 10% H_2_ and 10% CO_2_) at 37 °C. According to the growth curve of bacteria, the exponentially growing cells were harvested by centrifugation and diluted in BHI or MRS to 10^6^ CFU/mL based on a regression line derived from McFarland turbidity standards (Pro-Lab Diagnostics, Richmond Hill, ON, Canada).

### Adsorption assay of peptides

The amount of peptides adsorbed to the HAp disk surface was determined by fluorescent labelling technology^23^. HAp disks were pre-incubated respectively with 100 μL of fluorescein isothiocyanate (FITC) labelled peptides (FITC-KSLW and FITC-HBAMP) at the same molar concentration (92.60 μmol/L, 2× MIC of KSLW for *S. mutans*) for 2, 5, 10, and 20 min. Then the disks were washed thrice with sterile deionized water and observed under confocal laser scanning microscope (CLSM, OLYMPUS FV500, Tokyo, Japan) at excitation wavelength of 488 nm. Images were obtained with a 20× objective and at least three representative images were collected at different sites from each sample.

### Antibacterial activity against planktonic bacteria: Minimal inhibitory concentration (MIC) and minimal bactericidal concentration (MBC)

MIC and MBC assays were conducted according to the consensus guidelines developed by the Clinical and Laboratory Standards Institute (CLSI)^24^. Two-fold serial dilutions of peptides were prepared in culture medium at a volume of 200 μl/well in 96-well plates and the final concentrations of peptides ranged from 31.25 μg/ml to 2000 μg/ml. Chlorhexidine (0.12%) and culture medium were included as the positive and control group, respectively. Then each well was seeded with 20 μL of bacterial cell suspension (10^6^ CFU/mL). The plates were incubated anaerobically at 37 °C for 24 h. The MIC was defined as the lowest concentration of peptide that prevents visible growth of the bacteria tested^25^. To determine the MBC, 100 μL aforementioned contents of each well was respectively spread on agar plates and grown anaerobically at 37 °C for 24 h. The MBC was defined as the lowest concentration of peptide resulting in no colony formation on agar plates^26^. All determinations were made from triplicate experiments.

### Time-kill and growth inhibition curves assay

The time-kill kinetics of HBAMP against *S. mutans* was determined by time-kill assay as previously described^27^. *S. mutans* was grown to exponential phase and diluted to 10^6^ CFU/mL in growth medium. HBAMP was added into the tubes of bacterial suspension and the final concentration was 250 μg/ml (2× MIC) and 500 μg/ml (4× MIC). Bacterial suspension in other tubes that were added with 0.12% CHX and PBS served as the positive and blank control groups, respectively. At time points of 0, 10, 20, 30, 40, 60, and 90 min, 10 μL of cell suspension was collected, serially diluted in PBS and then 50 μL of aliquots were plated onto BHI agar plates. Colony forming units were counted after the plates were incubated anaerobically at 37 °C for 24 h.

The effect of the peptide on the growth of *S. mutans* was examined by a method similar to the above with a slight modification: The final concentration of HBAMP in the bacterial suspension was adjusted to 125 μg/ml (1× MIC). PBS was used as control group. At time points of 0, 20, 40, 60, 120, 180, 240, 480, and 720 min, 10 μL of cell suspension was collected, serially diluted in PBS and then 50 μL aliquots were plated onto BHI agar plates. Colony forming units were counted after the plates were incubated anaerobically at 37 °C for 24 h. All determinations were made from triplicate experiments.

### Antibacterial activity of HBAMP after binding to HAp: Biofilm viability on HBAMP coated HAp

The HAp disks, pre-incubated respectively with 100 μL of HBAMP and KSLW (92.60 μmol/L) for 5 min, were placed in 24-well plate. Each well of the plate was inoculated with 2 ml of *S. mutans* in BHIS (10^6^ CFU/mL). After incubation anaerobically at 37 °C for 12 h, the HAp disks were washed twice with sterile deionized water. Then biofilms were stained for 20 min in the dark using the LIVE/DEAD^®^ BacLight^TM^ Bacterial Viability Kit (Molecular Probes, Eugene, OR, USA). The stained biofilms were scanned layer by layer, from top to bottom, by CLSM at excitation wavelengths of 488 nm (SYTO 9) and 568 nm (propidium iodidd, PI). The HAp disk surface without peptide coating was processed using the same protocol. Images were obtained with a 20× objective and at least three images were collected randomly from each sample.

### Anti-biofilm activity against biofilm that colonized on HAp surface

*S. mutans* biofilms were grown on the surface of HAp disks that were placed in 24-well plate. Each well of the plate was inoculated with 2 ml of *S. mutans* in BHIS (10^6^ CFU/mL). After incubation anaerobically at 37 °C for 12 h, the HAp disks were washed twice with sterile deionized water and then respectively treated with HBAMP (2× and 4× MIC) and CHX (0.12%) for 10 min. Then biofilms were stained for 20 min in the dark using the LIVE/DEAD^®^ BacLight^TM^ Bacterial Viability Kit. The biofilm without treatment served as control. Stained biofilms were photographed by CLSM with a 20× objective. Three independent biofilm experiments were performed.

### Stability of HBAMP in human saliva

The protocols in this section were approved by the Ethics Committee of Zhongshan Hospital, Xiamen University. The methods employed were performed in accordance with the approved guidelines. After acquiring informed consent, human saliva was collected from individual volunteers (two males and two females; Minimum age: 22; maximum age: 28) in the morning prior to oral cleaning. The collected saliva samples were centrifuged at 12,000 rpm for 20 min. The supernatant was collected and filtered through the 0.45 mm membrane filter to remove any debris. HBAMP was added into the human saliva to achieve the final concentration of 500 μg/ml. The saliva with HBAMP was incubated at 37 °C. At 0, 5, 20 and 60 min, 1 ml samples were taken from the total sample and served in the specific sample bottles, the machine will automatically extract samples from the bottles to detect. The samples were analysed by reversed-phase HPLC using Zorbax Eclipse XDB-C18 analytical column (150 × 4.6 mm, Agilent Technologies, Inc., Santa Clara, CA, USA) protected by a XDB-C18 guard column (4 × 4 mm). For the elution of HBAMP, a flow rate of 1.2 mL/min and a linear gradient from 88:12 to 65:35 (0.1% TFA in water: 0.1% TFA in acetonitrile) for 10 min were employed. Total run time of HPLC-UV (215 nm) analysis was 15 min and the injection volume was set at 40 μL.

### Cytocompatibility tests

HBAMP should have negligible toxicity to human cells before it can be used in oral environment^28^. Human gingival fibroblasts (HGFs) were incubated in Dulbecco’s modified Eagle’s medium (GIBCO, Grand Island, NY, USA) containing 10% newborn calf serum (GIBCO, Grand Island, NY, USA) and 1% penicillin-streptomycin (GIBCO, Grand Island, NY, USA)^29^. Passage 6 cultures were used for experimental test. HGFs (2 × 10^3^) were seeded to each well of the 96-well plates and cultured for 48 h. Then cells were exposed to HBAMP at two different concentrations: 250 μg/ml and 500 μg/ml. Medium was used as a non-treated control. After 10, 60 and 240 min treatment in an incubator at 37 °C, the test cultures were centrifuged. The harvested cell cultured for another 24 h with 200 μl of fresh medium. The proliferation of HGFs after exposure to HBAMP was evaluated by Cell Counting Kit-8 (Beyotime, Haimen, China). The result was read at 450 nm using a microplate reader (Bio-Rad Lab, Hercules, CA, USA). The supernatants at designated time points were transferred to a new plate. Quantitation of lactate dehydrogenase (LDH) that was released into culture medium was performed with the Pierce^TM^ LDH Cytotoxicity Assay Kit (Thermo Scientific, Pittsburgh, PA, USA). Briefly, the supernatants containing extracellular LDH were mixed with Reaction Mixture. After incubation for 30 min, the reaction was stopped by adding Stop Solution. Absorbances at 490 nm were measured using a microplate reader (Bio-Rad Lab, Hercules, CA, USA). LDH leakage was expressed as the percentage (%) of the total LDH activity, where the total LDH activity of the cells was determined by exposing them to 0.2% (v/v) Triton X-100.

### Statistical analysis

The results were analysed with GraphPad Prism software (GraphPad Software, San Diego, CA, USA). Statistical analyses were performed by SPSS 19.0 software (IBM Corporation, Armonk, NY, USA) at a significance level of P < 0.05. ANOVA was used to examine the inter-group differences and the Tukey HSD tests were used to compare individual groups.

## Results

### Visualization of immobilized peptides

**Figure 1:**
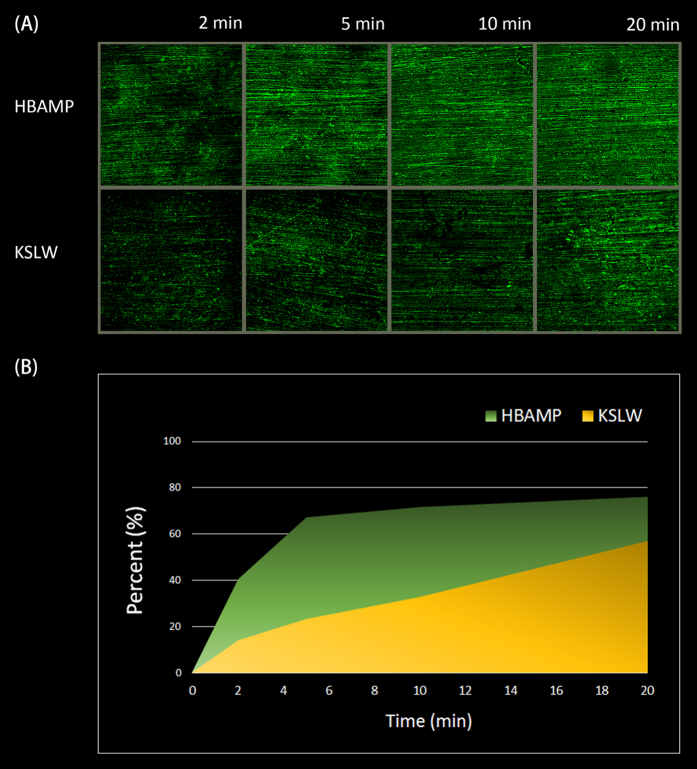
CLSM images of FITC-HBAMP and FITC-KSLW bound to HAp disk surface. (**A**) At the same molar concentration, the stronger fluorescent intensity on the surface pre-coated with HBAMP than the surface pre-coated with KSLW at the same time. (**B**) The relative amounts of fluorescent pixels at various time points.

HBAMP and KSLW were conjugated with fluorescein (FITC) to permit direct visualization of the peptides immobilized on HAp disk surface by using CLSM. As shown in Fig. 1, for each incubation time, the coverage rate of HBAMP on HAp disk surface was higher than that of KSLW at same molar concentration. Within 5 min, HBAMP could cover 67.3% of the HAp disk surface while KSLW covered 23.3%.

### Antibacterial activity against planktonic bacteria: MIC and MBC

The concentrations of HBAMP required to inhibit or to kill planktonic bacteria are summarized in Table 1. MIC values ranged from 125 to 500 μg/ml and MBC values ranged from 250 to 2000 μg/ml. Among the tested bacterial, *S. mutans* and *L. acidophilus* were most susceptible to HBAMP, with MIC values of 125 μg/ml. Except for *S. sanguinis*, MBC values of other tested bacteria were 2-fold higher than their MIC values. In addition, current results indicated that the antibacterial active site of HBAMP was KSLW.

### Time-kill and growth inhibition curves assay

**Figure 2:**
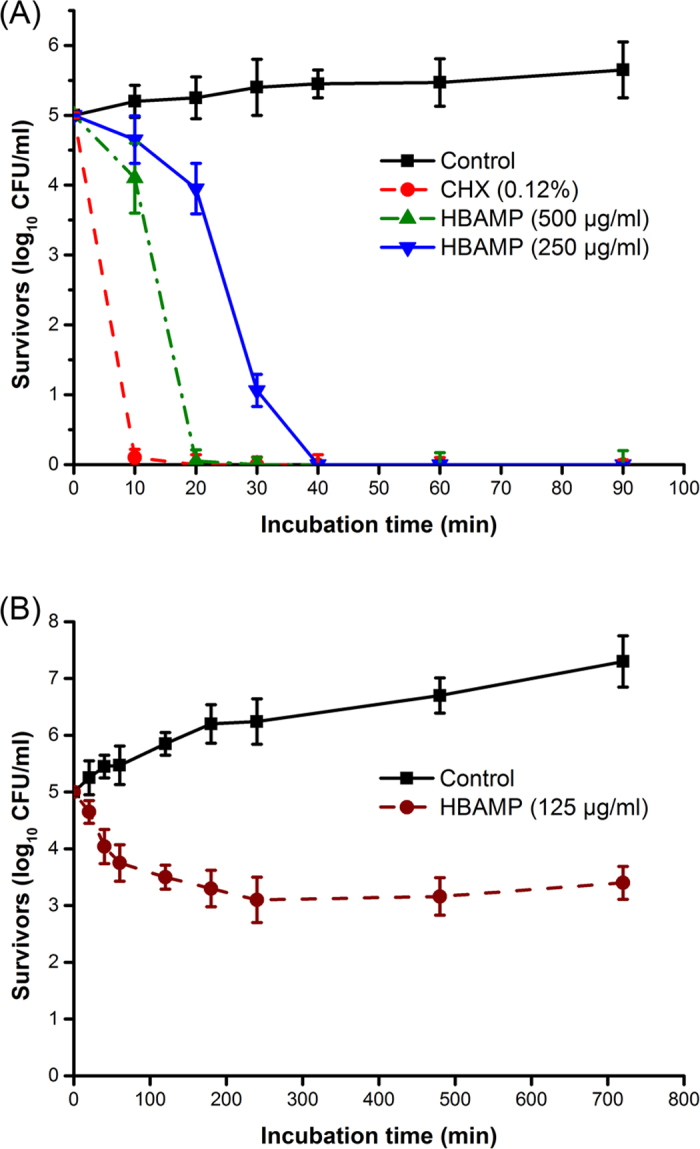
(**A**) Time-kill curves for *S. mutans* treated by HBAMP (250 μg/ml and 500 μg/ml) and CHX (0.12%). (**B**) Time-inhibit curves for *S. mutans* treated by HBAMP at 125 μg/ml.

The kinetics for killing *S. mutans* is shown in Fig. 2A. HBAMP exhibited concentration-dependent and time-dependent bacterial killing characteristics. CHX (0.12%) showed faster bactericidal effect than HBAMP at 250 μg/ml and 500 μg/ml. CHX could kill *S. mutans* within 10 min, while 250 μg/ml HBAMP reduced the viable counts of *S. mutans* by more than one order of magnitude after 20 min incubation and no viable cells could be detected after 40 min and 500 μg/ml HBAMP could kill *S. mutans* within 20 min.

As shown in Fig. 2B, the number of viable cells decreased constantly within 240 min when HBAMP (125 μg/ml) was added to the exponentially growing *S. mutans* cultures. From 240 to 720 min, the growth rate of *S. mutans* was significantly lower than the non-treated control group.

### Antibacterial activity of HBAMP testing after binding to HAp: Biofilm viability on HBAMP-coated HAp

**Figure 3:**
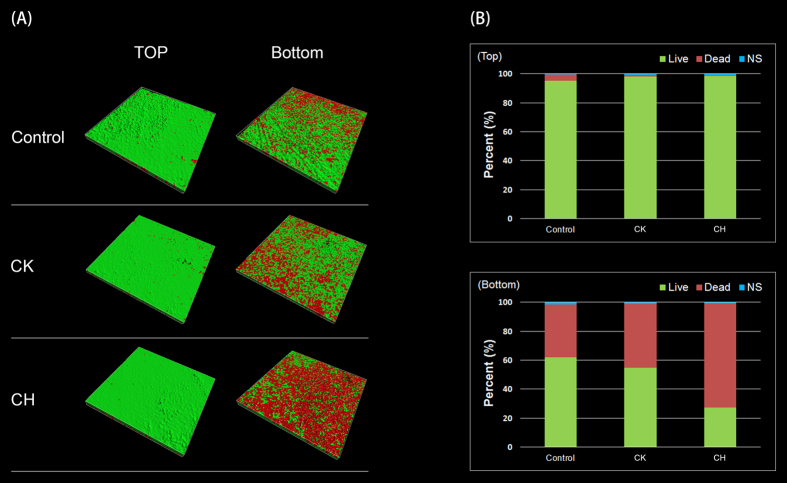
(**A**) CLSM images of top and bottom surface of *S. mutans* biofilm formed on the surface of HAp disks. Dead cells were stained red and live cells were stained green. (Control) *S. mutans* biofilm formed on untreated surfaces; (CK) *S. mutans* biofilm formed on the surfaces pre-coated with KSLW; (CH) *S. mutans* biofilm formed on the surfaces pre-coated with HBAMP. (**B**) The percentage of live/dead cells on the top and bottom surface of *S. mutans* biofilm.

The *S. mutans* biofilms generated on the HAp disk surface which pre-coated respectively with HBAMP and KSLW were shown in Fig. 3. The live bacteria were stained by SYTO 9 showed green fluorescence, whereas the dead bacteria were stained by propidium iodide showed red fluorescence. Compared with the untreated control, KSLW and HBAMP pre-coated had little impact on the viability of cells on the top surface of biofilm (97.4% viability on average). In addition, the viability of cells on the bottom of the biofilm was not statistically different between the untreated and KSLW pre-coated (P > 0.05; 58.3% viability on average). However, compared with the untreated control and KSLW pre-coated, green signals significantly reduced on the bottom of the biofilm when disks surface pre-coated with HBAMP (27.1% viability), suggesting that part of the cells in the bottom of biofilm were killed by the HBAMP.

### Anti-biofilm activity against biofilm that colonized on HAp surface

**Figure 4:**
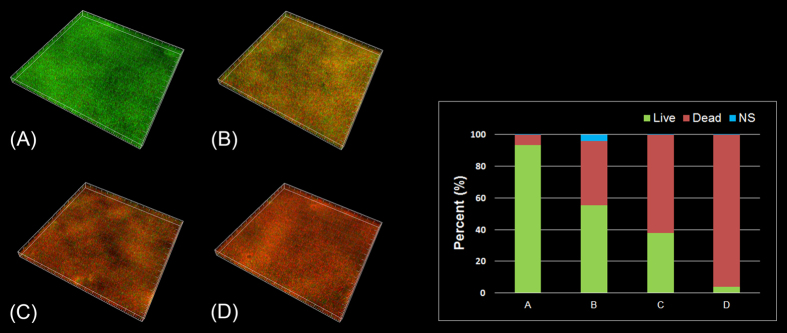
CLSM images of *S. mutans* biofilms and the percentage of live/dead cells. The biofilms were cultured for 12 h and then treated with 2×MIC HBAMP (**B**), 4× MIC HBAMP (**C**) and 0.12% CHX (**D**) for 10 min. The biofilm without treatment were performed by the same process as control (**A**).

The images of *S. mutans* biofilms treated with HBAMP and Chlorhexidine are showed in Fig. 4. HBAMP showed concentration-dependent characteristic in biofilm killing. After *S. mutans* biofilms were treated by HBAMP (250 μg/ml and 500 μg/ml, respectively) for 10 min, approximately 44.7% and 65.0% bacteria were killed, respectively. While about 96.2% bacteria were killed with the usage of CHX (0.12%) treating *S. mutans* biofilms for 10 min. The non-treated control displayed mostly green fluorescence, with a cell viability of 93.4%. There were significant differences between the treated groups and the non-treated control group (P < 0.05).

### Stability Study

**Figure 5:**
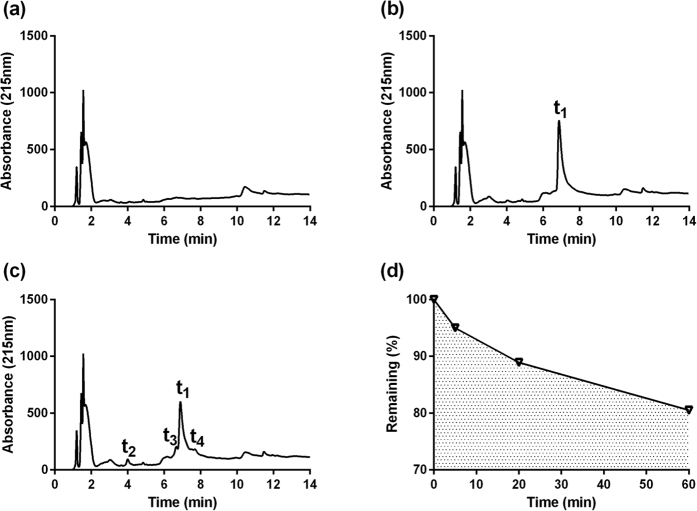
(**a**) HPLC chromatograms of human saliva; (**b**) Human saliva spiked with HBAMP (t1 represented the HBAMP, T = 6.9 min); (**c**) HBAMP (500 μg/ml) incubated in human saliva at 37 °C for 60 min (t2, t3, t4 represented degradation product of HBAMP, Time = 4.0, 6.6 and 7.6 min). (**d**) Degradation rate of HBAMP in human saliva at 37 °C in 60 min.

The HPLC chromatograms obtained from human saliva, human saliva mixed with HBAMP, and HBAMP incubated in human saliva are showed in Fig. 5. The results demonstrated that HBAMP has desirable stability in human saliva, while 80.5% of HBAMP (the initial concentration was 500 μg/ml) was intact after incubated in human saliva at 37 °C for 60 min.

As shown in Fig. 5c, while HBAMP incubated in human saliva, three degradation products, resolved from intact HBAMP (Time = 6.9 min), were detected at the time of 4.0, 6.6 and 7.6 min.

### Viability of HBAMP treated HGFs

**Figure 6:**
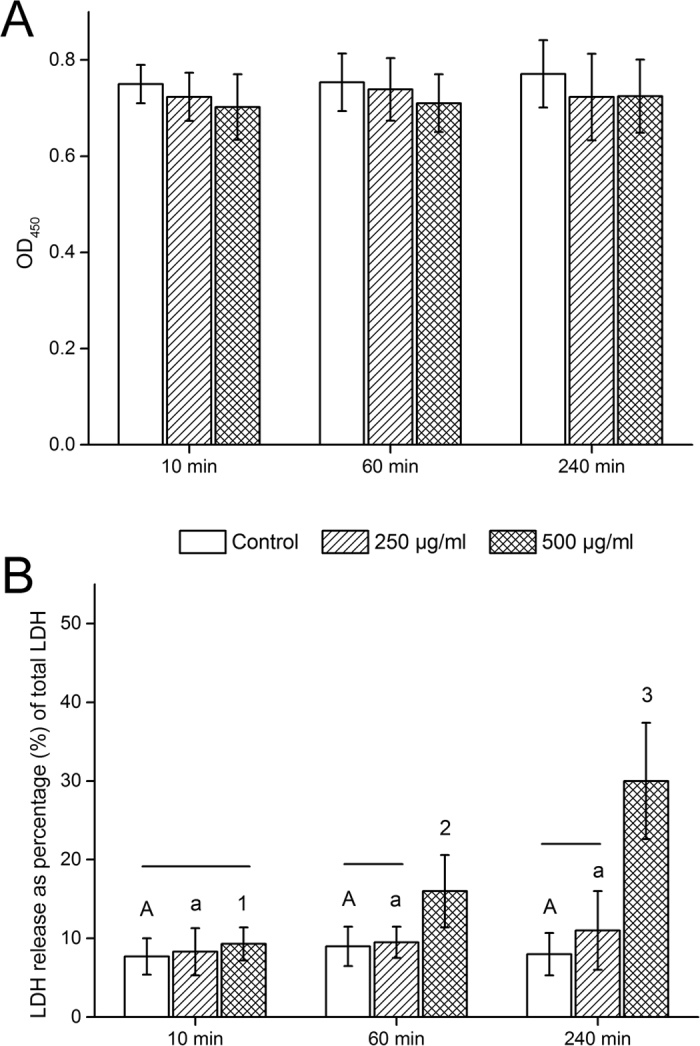
(**A**) Evaluation of the proliferation of HGFs treated with HBAMP at 250 and 500 μg/ml for 10, 60 and 240 min by CCK-8 assays. No difference was detected between all groups (P > 0.05). (**B**) Quantitation of lactate dehydrogenase (LDH) release of HGFs treated with HBAMP at 250 and 500 μg/ml for 10, 60 and 240 min by Pierce^TM^ LDH Cytotoxicity Assay Kit. For the factor “exposure time”, groups labelled with the same designators (upper case letters for control, lower case letters for 250 μg/ml, and numbers for 500 μg/ml) are not significantly different (P > 0.05). For the factor “HBAMP concentration”, groups from the same exposure time that are connected with a horizontal bar are not significantly different (P > 0.05).

As shown in Fig. 6A, HBAMP had little effect on proliferation of HGFs at concentrations of 250 and 500 μg/ml for 10, 60 and 240 min of incubation, with an optical density ranging from 0.7 to 0.8. There were no significant differences between the HBAMP-treated groups under the abovementioned conditions and the non-treated control groups (P > 0.05). Figure 6B shows that exposure to 250 μg/ml of HBAMP up to 240 did not affect membrane integrity of HGFs (P > 0.05), while 500 μg/ml of HBAMP exposure caused LDH leakage in a time dependent manner (P < 0.05).

## Discussion

**Figure 7:**
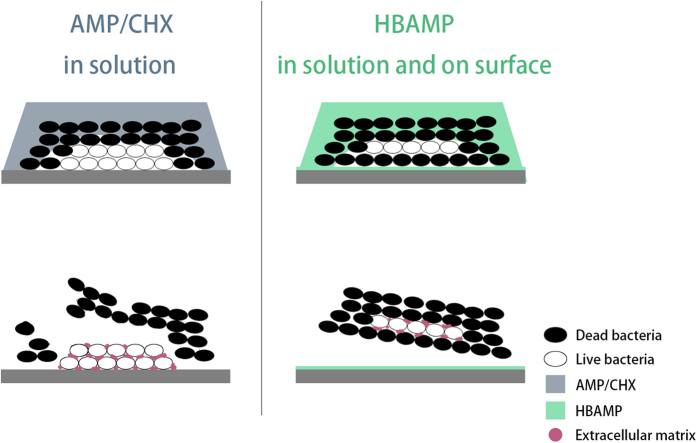
Schematic illustration of anti-biofilm mode of free AMP or CHX in solution (left) and synergistic anti-biofilm activity of HBAMP free in solution and bound on surface (right).

The prevention of oral infectious diseases such as periodontitis and caries was targeted at the control of the pathogenic biofilm formation on tooth surface. Studies have shown that conventional antibiotics, such as chlorhexidine, phenolic compounds, etc., could inhibit the formation of biofilms by killing bacteria directly and affecting the bacterial metabolism. However, they are not suitable for long-term use in practice, as they may cause tooth staining, drug resistance or other significant side-effects. AMPs exhibit broad-spectrum antimicrobial effect against a wide spectrum of bacterial species, including drug-resistant strains^30^,^31^,^32^, appearing as promising antibiotic surrogates. But their application in the oral cavity was limited due to the rapid dilution and enzymatic degradation by human saliva. Current study reports a novel molecular bioconjugate, i.e., HBAMP, consisting of a broad-spectrum antimicrobial peptide and a HAp-binding heptapeptide domain. It represents a technical advance in the field of oral biofilm control with three important merits: 1, it improves the retention of antimicrobial agents for oral use by adhering to HAp surfaces without losing its antibacterial activity; 2, it can form a contact-active antibacterial barrier on tooth surface to inhibit the formation of biofilms; 3, HBAMP free in solution and bound on HAp surface could act synergistically to reduce biofilm viability (Fig. 7), thus preventing the biofilm-associated oral diseases.

Studies by others have demonstrated that the silanised HAp surface could be covalently linked with peptides bearing desired functionality^33^,^34^. Their results suggested that the silanisation procedure is an efficient method to immobilize bioactive molecules on the materials surface. However, silanisation might alter the HAp surface markedly, and the surface must be silanised again when linking new antimicrobial peptides, which was not suitable in the environment of oral cavity. In the oral cavity, if teeth were not strictly separated from saliva, the silanised surface may covalently link with salivary proteins which exist in saliva rather than the antimicrobial peptides. In this study, we conjugated AMP with a HAp-binding heptapeptide (i.e., HBP7) domain, which can increase AMP binding to HAp surface through electrostatic attraction. This is a promising surrogate to silanisation and would not have a significant impact on the HAp surface. The HAp-binding domain of HBAMP was isolated from a linear 7-mer peptide phage display library, by using enamel as binding substrate^20^. The binding affinity of HBP7 with enamel was evaluated using Output/Input affinity test according to a standard protocol developed by the manufacture (New England Biolabs, Ipswich, MA, USA). This binding affinity of HBP7 over enamel surface was also verified by CLSM analysis. It was found that HBP7 binds onto the enamel surface with higher affinity, comparing to the affinity of HBP7 to enamel longitudinal section. It was proposed that electrostatic interaction between HBP7 and HAp plays a critical role in the binding process of HBP7 with HAp. Since HBP7 was identified through phage display library using enamel as binding substrate, we believe that HBAMP would bind to enamel with high affinity as long as the enamel surface is cleaned by toothbrushing. Since KSLW is a cationic molecule, it may have electrostatic attraction effect with HAp. However, our results showed only small amounts of KSLW bind to HAp disk surface compared to HBAMP at 5 min at the same molar concentration. Therefore, it is believed that HBP7 may play an important role in increasing specific binding affinity of HBAMP to HAp.

Our current study demonstrated that the free HBAMP has desirable antibacterial activity against oral planktonic bacteria, with *S. mutans* and *L. acidophilus* being the most sensitive species (MICs = 125 μg/ml). Time kinetics assays showed that 500 μg/ml HBAMP could kill planktonic *S. mutans* within 20 min. The reversed-phase HPLC chromatograms showed 500 μg/ml HBAMP was degraded smoothly and 80.5% remained after 60 min (Fig. 5d), which is in line with a previous study on the KSLW stability^16^. These data suggested that HBAMP not only has strong enough antimicrobial activity, but was stable enough when dissolved in saliva. These were essential for HBAMP to exert long-time antimicrobial effectiveness.

**Figure 8:**
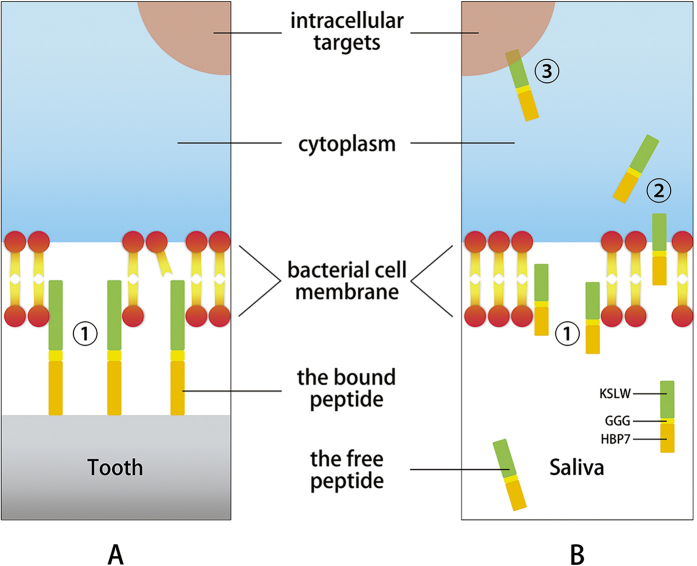
Schematic illustration of molecular mechanisms underlying the antimicrobial activity of HBAMP. HBAMP may damage bacterial cell membrane by disrupting lipid bilayer➀, translocate into cell interior➁, and interact with intracellular targets, which result in regulation of certain genes that control growth, transition, and biofilm formation➂. (**A**) HBAMP bound on tooth surface; (**B**) HBAMP free in saliva.

The general antibacterial peptide could only infiltrate inwards from the surface of the biofilm to kill bacteria gradually, and it usually failed to kill the bacteria at the bottom of biofilm. Hence, contact-active antibacterial surfaces are promising in inhibiting formation of biofilm on tooth surface^35^. Observed by CLSM, a significant increase in dead bacteria was identified at the bottom of biofilm which colonized on the HBAMP pre-coated HAp surface. Various mechanisms were proposed on the antibacterial activity of AMPs, including the formation of pores, disintegration of the membrane bilayer and attacking the cytoplasm and metabolic functions of the bacteria^36^. It was initially demonstrated by Hong *et al*. that KSL peptides may kill micro-organisms by attacking their membranes^37^. By using a BacLight^TM^ Bacterial Viability Kit, current study showed that HBAMP could cause bacterial cell membrane damage, as proved by the presence of a significant number of bacterial cells that are red-fluorescence (i.e., PI) stained within the HBAMP-treated *S. mutans* biofilm. Other studies also imply that HBAMP may act on bacterial intracellular targets that result in modulation of certain gene expression^38^. The initial interaction between the bacteria and AMPs is electrostatic because AMPs are cationic, which renders a strong interaction with the negatively charged bacterial membranes, penetrating the cell membrane, and acting on intracellular targets to inhibit cellular functions^39^. However, how the AMPs that are tethered to material surface through another artificial peptide domain damage and kill microorganisms was not fully understood. There are three possible explanations for the mechanism of reducing viable cells on the HAp surfaces pre-coated with HBAMP. The first possible mechanism is the direct antimicrobial activity of the bound HBAMP; the second is the action of the free HBAMP; and the third possible mechanism is the effective antimicrobial peptide domain that contains KSLW released from HBAMP bioconjugate by enzymatic degradation (Fig. 8). However, further study is needed to verify the facticity of these mechanisms. Contact-active antibacterial surfaces are often considered to be self-deactivating, because killed bacteria may adhere to the bioactive surface and new approaching bacteria can adhere and proliferate on these corpses. However, in the oral environment, killed bacteria at the bottom of the biofilm was unable to secrete extracellular matrix, thereby reducing the adhesion of biofilm on the surface of the teeth. The bacterial corpses can be easily removed from the surface of the teeth through oral self-cleaning capability, normally provided by speaking, chewing, and appropriate salivation (Fig. 7).

The sessile bacterial cells within biofilm are more resistant to the antimicrobial agents than planktonic cells^40^. CHX has broad antibacterial effect and is most widely used in commercial mouth rinses^41^. While AMP could decrease the formation of biofilms by inhibiting or killing planktonic bacteria, it also bears the functionality to kill bacteria in biofilm^42^. In this study, *S. mutans* biofilms on HAp surface were treated with HBAMP (2× and 4× MIC) and CHX (0.12%) for 10 min. HBAMP at 4× MIC could kill approximately 40% of the bacteria in 10 min, with 0.12% CHX kill approximately 95%. This result suggests that CHX might have the ability to penetrate deeper into the biofilms than the free HBAMP. Although the free HBAMP could not eradicate all *S. mutans* cells within the biofilms, HBAMP that adhered to the HAp surface can form a contact-active antibacterial surface, which can kill bacteria that came into contact^43^.

## Conclusions

In summary, the results of this study indicate that HAp surface modification using HBAMP, a molecular bioconjugate consisting of a broad-spectrum antimicrobial peptide and a specific HAp-binding peptide, is a feasible approach to inhibit biofilm formation on tooth surfaces. Our *in vitro* experiments demonstrated that HBAMP can rapidly bind to the HAp surface to form a contact-active antibacterial surface. It is cytocompatible to HGFs and bears desirable stability in human saliva. While the mechanism of this contact-active antibacterial surface remains to be further clarified, current study provides a proof-of-concept on using conjugated molecules to reduce biofilm formation by synergistically action of antibacterial agents free in solution and bound on surface.

## Additional Information

**How to cite this article**: Huang, Z.- *et al*. Design of a hydroxyapatite-binding antimicrobial peptide with improved retention and antibacterial efficacy for oral pathogen control. *Sci. Rep.* **6**, 38410; doi: 10.1038/srep38410 (2016).

**Publisher's note:** Springer Nature remains neutral with regard to jurisdictional claims in published maps and institutional affiliations.

## Figures and Tables

**Table 1:** Antibacterial activity of the HBAMP and its constituent peptides against oral microorganisms^1^.

Peptide	Amino acid Sequence	*S. mutans*		*S. sanguinis*		*L. acidophilus*		*A. viscosus*	
		MIC	MBC	MIC	MBC	MIC	MBC	MIC	MBC
HBP7	NNHYLPR	N^2^	N	N	N	N	N	N	N
KSLW	KKVVFWVKFK	62.5	125	250	2000	62.5	125	125	500
HBAMP	NNHYLPRGGG- KKVVFWVKFK	125	250	500	2000	125	250	250	500

^1^MICs and MBCs are shown in microgram per milliliter (μg/ml).

^2^The bacteriostatic activities were not apparent under all concentrations tested.
